# Direct MinE–membrane interaction contributes to the proper localization of MinDE in *E. coli*

**DOI:** 10.1111/j.1365-2958.2009.07006.x

**Published:** 2009-12-24

**Authors:** Cheng-Wei Hsieh, Ti-Yu Lin, Hsin-Mei Lai, Chu-Chi Lin, Ting-Sung Hsieh, Yu-Ling Shih

**Affiliations:** 1 Institute of Biological Chemistry, Academia Sinica128 Sec 2, Academia Road, Nankang, Taipei 115, Taiwan; 2 Institute of Biochemical Sciences, National Taiwan University1 Sec 4 Roosevelt Road, Taipei 106, Taiwan

## Abstract

Dynamic oscillation of the Min system in *Escherichia coli* determines the placement of the division plane at the midcell. In addition to stimulating MinD ATPase activity, we report here that MinE can directly interact with the membrane and this interaction contributes to the proper MinDE localization and dynamics. The N-terminal domain of MinE is involved in direct contact between MinE and the membranes that may subsequently be stabilized by the C-terminal domain of MinE. In an *in vitro* system, MinE caused liposome deformation into membrane tubules, a property similar to that previously reported for MinD. We isolated a mutant MinE containing residue substitutions in R10, K11 and K12 that was fully capable of stimulating MinD ATPase activity, but was deficient in membrane binding. Importantly, this mutant was unable to support normal MinDE localization and oscillation, suggesting that direct MinE interaction with the membrane is critical for the dynamic behavior of the Min system.

## Introduction

The Min system forms a dynamic oscillator that drives the midcell placement of the cell division septum during cell cycle progression in *E. coli* (Lutkenhaus, 2008). MinD and MinE are the core components for sustaining the oscillation cycles. An exogenous supply of ATP can regenerate these cycles in the form of planar traveling waves on the membrane surface *in vitro* (Loose *et al.*, 2008). MinD directly associates with the cell membrane through the formation of an amphipathic helix at its extreme C-terminus upon binding with ATP (Szeto *et al.*, 2002; Zhou and Lutkenhaus, 2003). Upon membrane association, MinD assembles into long-range filamentous structures that wrap around the cell cylinder (Shih *et al.*, 2003). Recruitment of MinE via the membrane-associated MinD further stimulates MinD ATPase activity (Hu and Lutkenhaus, 2001), which in turn regulates the initiation of the oscillation cycles.

Previous reports mapped the MinD-interacting domain of MinE to the N-terminal amino acids 1–31 (MinE^1–31^) (Zhao *et al.*, 1995). This domain was predicted to form a nascent helix in solution by nuclear magnetic resonance analysis (King *et al.*, 1999). Mutational studies on one face of the helical wheel projection of amino acids 1–35 supported the idea that this region forms a complementary surface for interaction with MinD (Ma *et al.*, 2003). Two-hybrid analyses and structural modelling of putative MinD homologues indicated that the corresponding location in MinD for this interaction is an α-helix (helix 7) (Ma *et al.*, 2004; Zhou *et al.*, 2005). The C-terminal domain of MinE (MinE^32–88^) was designated the topological specificity domain, and its production in high levels in wild-type *E. coli* induced minicelling (Zhao *et al.*, 1995). Although the topological function of MinE has been attributed to a diffusion-reaction mechanism by theoretical modelling studies (Kruse *et al.*, 2007), the molecular and biochemical basis for this mechanism is still incomplete.

In this study, we report several lines of evidence to support that direct MinE–membrane interaction is necessary for the proper function of the Min system. The N-terminal domain of MinE appears responsible for this interaction that involves electrostatic force. The same region was known for interaction with MinD to stimulate MinD ATPase activity. In the mutational studies, we identified a mutant MinE (R10G/K11E/K12E) that was capable of stimulating MinD ATPase activity comparable to the wild-type MinE but failed to associate with the membrane. This MinE mutant was unable to sustain localization and oscillation of the Min proteins, thus supporting the physiological relevance of the observed MinE–membrane interaction. The direct interaction between the N-terminal domain of MinE and the membrane raises a possibility that the topological specificity function is delivered by the N-terminal domain. The C-terminal domain of MinE may serve to control the anti-MinCD and topological specificity functions through sequestering the N-terminal domain and through its dimerization ability.

## Results

### MinE interacts with the membrane through its N-terminal domain

**Fig. 1:**
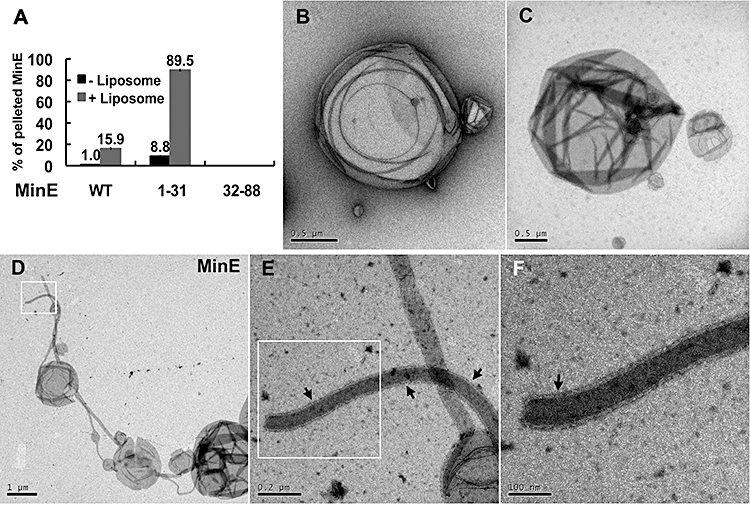
Evidence of direct MinE–membrane interaction. A. Quantitiative analyses of the percentage of pelleted full-length MinE, MinE^1–31^ and MinE^32–88^ with liposomes in the co-sedimentation assays. B–F. EM micrographs of free liposomes in solution (B and C) and MinE-associated tubules encircled by a coat (D–F; arrow). Micrographs (D–F) cover the same area and the boxed areas were viewed at higher magnification during acquisition. The width of this peripheral coat was approximately 9.4 nm.

In the current model, MinE is thought to associate with the membrane through recruitment by MinD. The significance of the direct MinE–membrane association is not confirmed. To verify the direct MinE–membrane interaction, we conducted *in vitro* sedimentation assays using purified MinE and liposomes reconstituted from *E. coli* polar lipids (Avanti). These showed that approximately 15.9 ± 0.9% of MinE segregated to the pellet fraction (Fig. 1A), suggestive of a direct interaction between MinE and the phospholipid bilayer. To better understand the protein domains of MinE responsible for this direct interaction with liposomes, we dissected MinE into N-terminal (MinE^1–31^) and C-terminal (MinE^32–88^) fragments. While MinE^32–88^ was completely absent in the pellet fraction (Fig. 1A), 89.5 ± 0.9% of MinE^1–31^ sedimented with liposomes. This result indicates that MinE^1–31^ is responsible for the MinE–membrane interaction and also that the membrane-binding ability of MinE is enhanced by removal of its C-terminal domain.

**Fig. 4:**
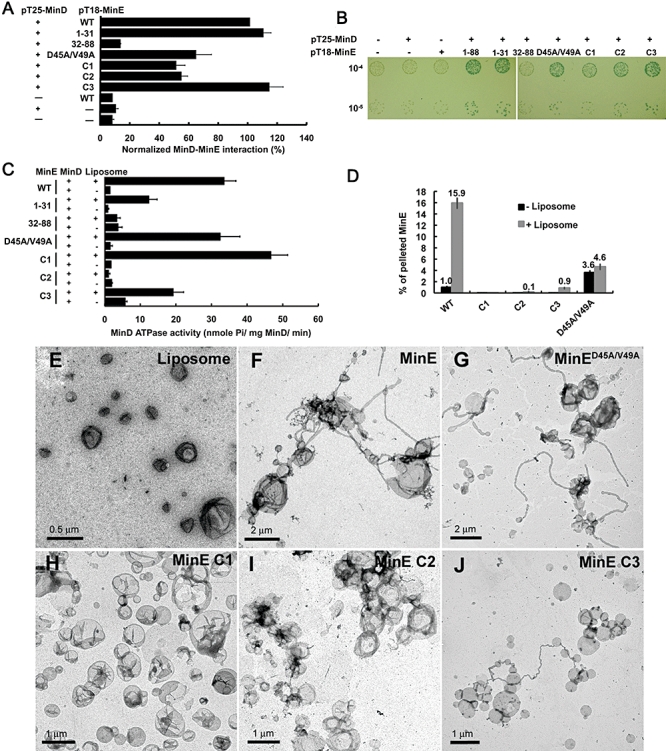
Abilities of the wild-type and mutant MinE proteins to interact with MinD, to stimulate MinD ATPase activity and to deform liposomes *in vitro*. A and B. The interaction between MinD and MinE was assayed using the bacterial two-hybrid system. Both liquid and plate assays are shown. Two dilutions (10^−4^, 10^−5^) of each culture were spotted on the same indicator plate (B). C. Stimulation of MinD ATPase activity by various MinE mutants in the presence or absence of liposomes. D. The full-length MinE carrying mutations in the N-terminal domain or in D45 and V49 did not co-sediment with liposomes. E–J. Degree of liposome deformation associating with the wild-type and mutant MinE proteins (C1, C2, C3 and D45A/V49A) was examined under EM.

Electron microscopy was performed to examine the same samples in the sedimentation assays. Purified MinE appeared as precipitates under the electron microscope. In contrast to the smooth outlines of liposomes presented alone in solution (Fig. 1B and C; Fig. 4E), there were tubules protruding out of the liposomes when MinE was added to the reactions (Fig. 1D–F; Fig. 4F). In some cases the membrane tubules were surrounded by a coat with a width greater than the phospholipid bilayer (Fig. 1E and F), suggesting that liposome deformation was associated with the presence of MinE. The electron microscopy observations therefore support the existence of direct MinE–membrane interaction.

### Positively charged residues in MinE^1–31^ are responsible for interaction with the membrane

The N-terminal MinE^1–31^ fragment contains eight positively charged residues with a theoretical pI value of 11.45, raising the possibility that electrostatic forces may participate in MinE–membrane interaction. The hydrophobic residues apparently do not play a primary role based on two reasons. First, the uneven distribution of the hydrophobic residues in MinE^1–31^ is insufficient to support formation of an amphipathic helix and is also unlikely to form a single transmembrane helix. Second, amino acid substitutions in L22 and I25 of MinE resulted in no interaction with MinD, but the mutant MinE was still localized to the cell envelope (Ma *et al.*, 2003). Mass spectrometry was used to determine the molecular weight of the MinE fusion protein (Fig. S1A) and showed a reduction of approximately 132 Da. Using N-terminal protein sequencing, we found that the N-terminal methionine residue was removed from MinE. This may be due to the action of methionine aminopeptidase, which has a tendency to excise N-terminal methionines followed by a residue with a small side-chain at the second position (Frottin *et al.*, 2006). There were no additional signs of modification that could potentially support the protein–membrane interaction.

**Fig. 2:**
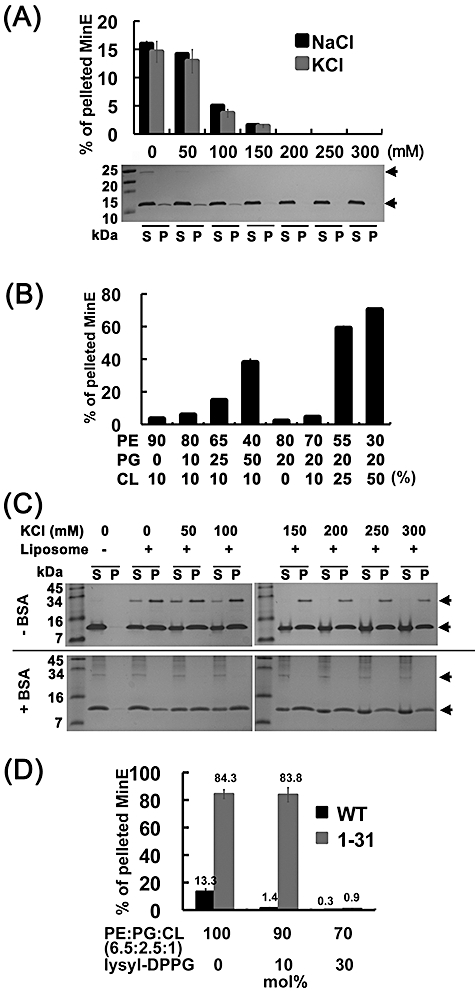
Electrostatic force is involved in mediating the MinE–membrane interaction. A. The co-sedimentation of full-length MinE with liposomes was disrupted by increasing concentrations of NaCl and KCl. The Tris-Tricine-SDS gel image exemplified an experiment with different NaCl concentrations that were used in the analyses. The lower arrow indicates the position of MinE. The higher arrow indicates an additional 28 kDa band induced by the presence of liposomes. S, supernatant; P, pellet. B. MinE showed a binding preference for the anionic phospholipids PG and CL. C. The MinE–membrane interaction was stabilized under high salt conditions by the presence of 50 mol% cardiolipin (PE : PG : CL = 36:14:50). The lower and higher panels show experiments with or without the presence of 18 µM BSA respectively. MinE was supplied at 6 µM in these reactions. Note that the 28 kDa band was relocated to the supernatant fraction in the presence of BSA, indicating its appearance in the pellet fraction was mostly non-specific. The additional bands in the lower gel were impurities of the BSA solution. D. Addition of cationic lysyl-DPPG to liposomes interfered with the co-sedimentation of MinE and MinE^1–31^ with liposomes. The reactions were incubated for 60 min prior to centrifugation.

We therefore investigated whether the N-terminal domain of MinE might interact with the head groups of the anionic phospholipids under an assumption that MinE attaches to the membrane through electrostatic forces that may in turn trigger conformational changes in the protein to stabilize the protein–membrane interaction. In agreement with the proposition that a charge interaction is involved, the amount of pelleted MinE gradually decreased with increasing salt concentrations (0–300 mM) in the sedimentation assays. In the presence of 200 mM KCl or NaCl, MinE completely failed to sediment (Fig. 2A). We then reconstituted liposomes of defined composition to examine the influence of different phospholipids on MinE sedimentation. As shown in Fig. 2B, the amount of pelleted MinE increased when the percentage of either phosphatidylglycerol (PG) or cardiolipin (CL) was increased in the liposome formulas. Raising the CL concentration had a stronger enhancement on MinE pelleting than raising the PG content. Furthermore, we found that the presence of 50 mol% cardiolipin not only increased the binding ability of the full-length MinE to liposomes, but also became resistant to the high salt conditions (Fig. 2C). Protein extracted from the pellet fraction in the sedimentation assay using liposomes containing 50 mol% cardiolipin was analysed by mass spectrometry. There were two major molecules identified at 11 168.1 and 22 336.1 Da that correlate to the monomeric and dimeric form of MinE (Fig. S1B).

In the presence of BSA, serving as a non-specific membrane-binding agent in the reactions, MinE preserved its ability to interact with liposomes even under high salt conditions (Fig. 2C). A slight reduction of pelleted MinE was only observed when the concentration of KCl was above 250 mM. In the absence of salt, non-specific BSA binding appeared to interfere with MinE binding to the membrane, but MinE sedimentation with liposomes was restored when 50 mM KCl or higher was supplemented in the reactions. These results suggested the observed interaction can occur under physiological conditions especially at the cardiolipin-enriched sites on the membrane.

In contrast to anionic phospholipids, the addition of 10 or 30 mol% of the cationic lysyl-dipalmitoylphosphatidylglycerol (lysyl-DPPG) to the liposome formulation significantly reduced the sedimentation of both the full-length MinE and MinE^1–31^ (Fig. 2D). This result supports the idea that the positive charge at the N-terminal domain of MinE contributes to the observed MinE–liposome interaction.

### Membrane association of MinE^1–31^ is affected by mutations in the basic residues

Eight positively charged residues in MinE^1–31^ are clustered in three regions, hereafter referred to as Cluster 1 (C1: R10/K11/K12), Cluster 2 (C2: K19/R21) and Cluster 3 (C3: R29/R30/R31). To identify specific sites responsible for interaction with the membrane, we first assessed amino acid conservation in MinE^1–31^ from different bacterial species (Fig. S2A). Among homologues from 149 bacterial species (111 Gram-negative proteobacteria, 17 Gram-positive bacteria and 21 cyanobacteria), the C2 residues were the most conserved. The C1 residues were only highly conserved among Gram-negative bacteria, and the C3 residues displayed the least conservation. Only 4.7% of the searched sequences had theoretical pI values below 9, and 35.6% were below 10 (Fig. S2B). Analysis of the residue conservation and theoretical pI values implied that the positive charge in MinE^1–31^ may have an important function or functions.

**Fig. 3:**
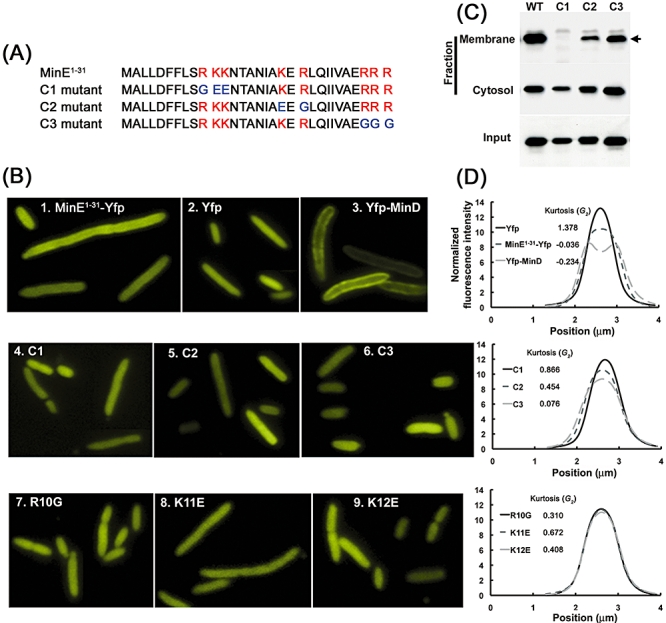
Membrane association of various mutants of MinE^1–31^-Yfp in Δ*min* cells. A. MinE^1–31^ contains three clusters of basic residues (red). This region of MinE^1–31^ was engineered to disrupt the positively charged residues in these clusters. The substituted residues are coloured in blue. B. Localization of wild-type (1) and mutant MinE^1–31^-Yfp (4–9) as well as untagged Yfp (2) and Yfp-MinD (3) in Δ*min* cells. C. Western blot analyses on fractionated cells expressing the mutant forms of MinE^1–31^-Yfp. The membrane fraction was estimated to be 30-fold more concentrated than the cytosolic fraction in this experiment to facilitate comparisons between different MinE^1–31^-Yfp bound to the membrane. The arrow indicates MinE^1–31^-Yfp. D. Analysis of fluorescence distribution across the cell width and *G_2_* of MinE^1–31^-Yfp, Yfp and Yfp-MinD (top), mutant MinE^1–31^-Yfp containing C1, C2 or C3 mutations (middle) and mutant MinE^1–31^-Yfp containing R10G, K11E or K12E mutations (bottom).

**Fig. 5:**
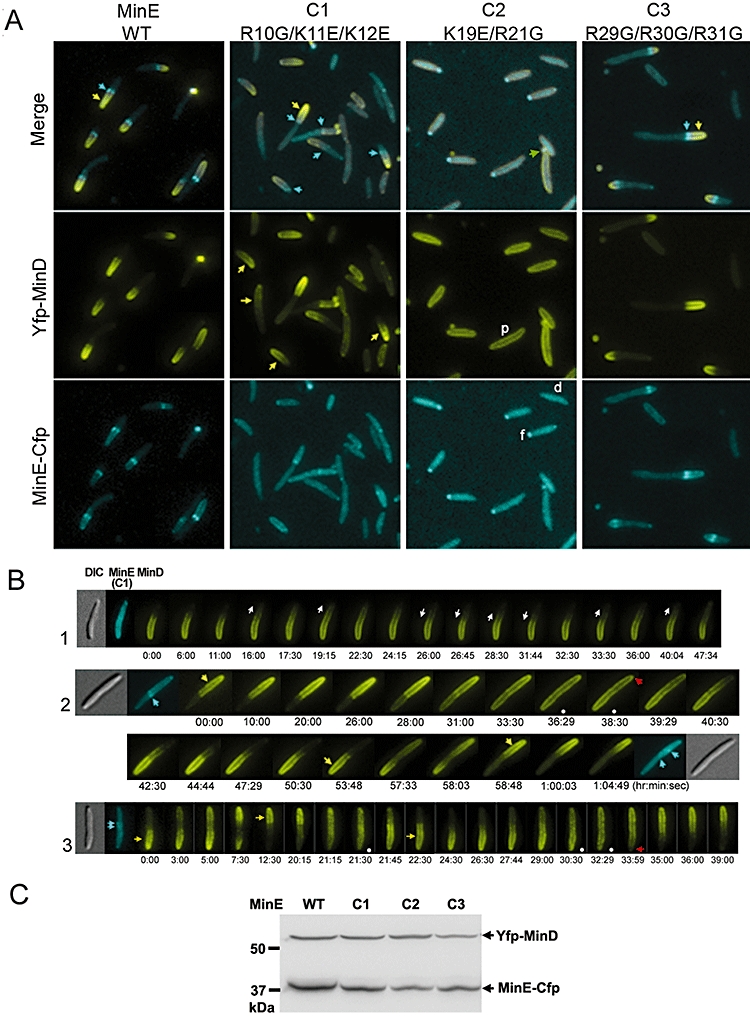
Effects of the N-terminal mutations on the localization of MinD and MinE. A. Colocalization of MinD with MinE lacking various positively charged residues (mutants C1, C2 and C3). p: peripherally localized MinD; d: diffusely distributed MinE; f: MinE focus. Yellow and cyan arrows indicate the MinD polar zones and the MinE rings respectively. The green arrow points out a fluorescent spot found in a minicell. B. Movement of MinD fluorescence in the presence of the C1 mutant MinE. The top cell started to disassemble MinD at one pole. White arrows indicate directions of polar zone growth and shrinkage. Another two cells showed atypical oscillation with a peculiar stage of MinD on the entire periphery of the cells (frames marked by the white dots) before re-initiating disassembly from the extreme cell pole (red arrows). C. Western blot analyses using the anti-Gfp antibody to detect levels of Yfp-MinD and MinE-Cfp in cells. The anti-Gfp antibody used here displayed different abilities to recognize Yfp and Cfp, thus the band intensity did not reflect the real ratio of Yfp-MinD to MinE-Cfp in cells.

**Fig. 6:**
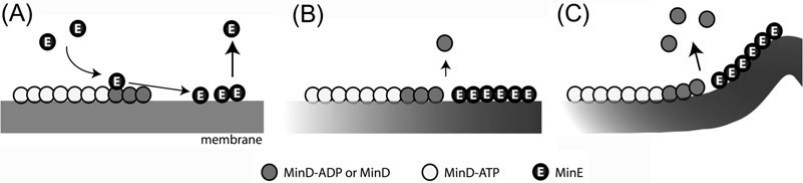
Model of MinE association with the membrane and a possible role of this membrane association in the Min system. A. The direct MinE–membrane interaction is facilitated at the membrane proximity through MinD's recruitment. However, this association is unstable on membranes where different phospholipids are randomly distributed. B. The MinE–membrane interaction can be stabilized by the presence of a high local concentration of cardiolipin at the midcell. A MinE ring subsequently appears at this site. The darker shade of the membrane represents the area enriched in cardiolipin. C. Three actions are required for dissociation of MinD from the membrane, including ATP hydrolysis in MinD, formation of the MinE ring, and MinE-induced membrane deformation. The degree of membrane deformation and the organization of MinE molecules in this process are under investigation.

To examine the importance of the positively charged residues, the cellular localization of MinE^1–31^-Yfp with mutations in the C1, C2 or C3 regions was examined in a *min*-deletion background (Fig. 3). Amino acid substitutions were introduced into the three clusters, respectively, to disrupt the positive charge (Fig. 3A). Consistent with a previous report (Ma *et al.*, 2003), wild-type MinE^1–31^-Yfp showed peripheral localization in Δ*min* cells (Fig. 3B, 1), but this localization was not as pronounced as that of the membrane-bound Yfp-MinD (Fig. 3B, 3). Because fluorescently tagged membrane-associated proteins can give rise to a visible boundary around the cell periphery, the clearness of the boundary can serve as an indicator to differentiate weakly or strongly bound membrane-associated proteins. With this criterion in mind, fluorescence of the C1 mutant construct appeared to be dispersed in cells (Fig. 3B, 4), similar to the fluorescence distribution of untagged Yfp (Fig. 3B, 2). The constructs harbouring single amino acid substitutions in the C1 region gave results similar to that of the triple C1 mutant (Fig. 3B, 7–9). The membrane association of MinE^1–31^-Yfp harbouring C2 or C3 mutations was less clear than that of the wild-type construct (Fig. 3B, 5, 6), however, it was not completely dispersed.

We fractionated cells carrying different MinE^1–31^-Yfp constructs to examine the degree of membrane association by Western blot analysis (Fig. 3C). We found that the C1 and the C2 mutant proteins were present at lower amounts in the input cell lysate, indicating these MinE^1–31^-Yfp mutant proteins were less stable in cells. In accordance with the fluorescence microscopy observations, the C1 mutant MinE was not detected in the membrane fraction, but a small amount of C2 mutant protein was present in the membrane. The C3 mutant construct was detected at a level less than the wild-type protein in the membrane fraction.

The degree of membrane association of MinE^1–31^-Yfp in Δ*min* cells was further assessed by the fluorescence intensity profile (Fig. 3D). The florescence intensity in a uniformly sized area across the cell width was measured in a population of cells (*n* = 50). The fluorescence intensity at each pixel position along the cell width was normalized as a percentage of the total fluorescence intensity in the area before obtaining the averages that was then plotted against its position (Fig. 3D). Kurtosis (*G_2_*), which is a measure of peakedness of a distribution in statistics, was calculated for each data series and was used to assist understanding the shape of the plot. Unlike the bimodally distributed intensity profile of Yfp-MinD (*G_2_* = −0.234), the profile of MinE^1–31^-Yfp formed a broad peak (*G_2_* = −0.036), while the profile of cytoplasmic Yfp formed a sharp peak (*G_2_* = 1.378). The C1 mutant yielded a profile similar to Yfp, and the other mutants showed broader peak distributions that fell in between those of Yfp and MinE^1–31^-Yfp. Calculation of *G_2_* for each data set by unbiased estimation revealed higher *G_2_* values for the C1 and C2 mutants (C1: 0.866; C2: 0.454; R10G: 0.310; K11E: 0.672; K12E: 0.408), indicating a higher probability of cytoplasmic distribution. The lower *G_2_* value of the C3 mutant construct (0.076) indicated a higher probability of membrane association. Thus both the fluorescence intensity profile and the Western blot analysis supported that the C1 residues contribute most significantly to the membrane association of MinE^1–31^-Yfp *in vivo*.

### The C1 mutant is a specific membrane-binding mutant

Confirmation of the membrane-associating property of the N-terminal domain of MinE raised a perplexing question about the interplay between the ability of MinE to interact with both the membrane and MinD. We investigated the ability of various MinE mutants to interact with MinD via bacterial two-hybrid assays (Fig. 4A and B); to stimulate MinD ATPase activity with ATPase assays (Fig. 4C); to co-sediment with liposomes (Fig. 4D); and to deform liposomes *in vitro* with electron microscopy (Figs 4E–J). A previous model stated that it is the N-terminal domain of MinE that stimulates MinD ATPase activity by interacting with MinD (Hu and Lutkenhaus, 2001). Consistent with this, the ATPase activity of MinD with MinE^1–31^ was approximately 36% of the wild-type level (WT: 33.1 ± 3.8 nmole Pi mg MinD^−1^ min^−1^; MinE^1–31^: 11.9 ± 2.8 nmole Pi mg MinD^−1^ min^−1^), but its activity with MinE^32–88^ was only about 9% of the wild-type level (2.9 ± 1.4 nmole Pi mg MinD^−1^ min^−1^). We were able to detect interaction between MinE^1–31^ and MinD at the same level as between the full-length MinE and MinD in the bacterial two-hybrid system, differing from a previous study that detected no interaction using the yeast two-hybrid system (Ma *et al.*, 2003). We suspect that this difference is due to the different assaying systems. It should be noted that the endogenous Min proteins in the bacterial two-hybrid system might have reduced the sensitivity of the system, but they did not interfere with the detected interaction, because only interactions between the fusion proteins could give rise to adenylate cyclase activity for downstream reactions. We concluded that the N-terminal domain of MinE is physically involved in the interaction with MinD and that the C-terminal domain is needed for MinE to fully stimulate the ATPase activity of MinD.

Assays with the three charge mutants of MinE provided further insights into the dual functions performed by the N-terminal domain of MinE. Briefly, the C1 mutant displayed weakened MinD–MinE interaction (approximate 50% reduction) but was enhanced for MinD ATP hydrolysis (40% increase). We reasoned that this was a consequence of more effective ATP hydrolysis, causing faster MinE dissociation from MinD that reflected on less stable MinD–MinE interaction. The C1 mutant also suggested that stimulation of the MinD ATPase activity does not involve direct MinE–membrane interaction. The C2 mutant reduced the ability of MinE to interact with MinD (44% reduction) and abolished ATP hydrolysis by MinD. The importance of these two residues in stimulation of MinD ATPase activity and interaction with MinD was reported previously (Hu and Lutkenhaus, 2001; Ma *et al.*, 2003). This observation is also in agreement with the fact that K19 and R21 are the most conserved charge residues among the MinE homologues from different species (Fig. S2A). The C3 mutant appeared to interact with MinD normally but had a weaker ability to stimulate the ATPase activity of MinD (43% reduction). It was previously proposed that the predicted α-helix of MinE^1–35^ may interact with residues D152 and S148 on helix 7 of MinD, leading to the release of K11 in the P-loop region for catalysing ATP hydrolysis (Ma *et al.*, 2004). Therefore, the C3 mutation of MinE may result in a failure to remove helix 7 of MinD from its position blocking the ATP catalytic domain. It should be noted that only a basal level of ATPase activity was detected from either wild-type or mutant MinE on its own (Fig. S3).

Through the *in vitro* sedimentation experiments, we found that all three mutant MinE proteins failed to cause significant sedimentation (Fig. 4D). The membrane-deforming properties of the MinE mutants were also examined under the electron microscope at lower magnification (Fig. 4E–J). While extended membrane tubules were frequently found with wild-type MinE in the reaction (Fig. 4F), membrane tubules were identified in fewer cases with the C3 mutant (Fig. 4J) and were rarely seen when both C1 and C2 mutants were tested (Fig. 4H and I). Taken altogether, only the C1 mutant was classified as a specific membrane-binding mutant (Table 1).

**Table 1:** Functions of the wild-type and mutant MinE proteins.

	MinE						
Function	WT	1–31	32–88	D45A/V49A	C1	C2	C3
Sedimentation with liposome	+	+++	−	−	−	−	−
Membrane deformation *in vitro*	+++	ND	ND	++	−	−	+
Interaction with MinD	++	++	−	+	+	+	++
Stimulation of MinD ATPase activity	++	+	−	++	+++	−	+
Oscillation (half cycle per minute)	+ (0.62)	−	NA	+ (aberrant)	+ (aberrant)	−	+ (0.19)

ND, not determined; NA, not applicable.

### Direct MinE–membrane interaction is required for proper localization of MinD and MinE *in vivo*

The physiological relevance of the direct MinE–membrane interaction was demonstrated by examining the effects of the MinE mutants on MinDE localization and oscillation in a *min*-deletion strain (YLS1) expressing the construct P*_lac_*::*yfp-minD minE-cfp* (Shih *et al.*, 2002a). With the C1 mutant of MinE, formation of the MinD polar zone and the MinE ring was found in a smaller proportion of cells, and the MinD polar zone often exceeded half of the cell length (Fig. 5A, Table 2). The phenotype of the C1 mutant resembled that of the previously described D45A/V49A mutant (Shih *et al.*, 2002a), but the frequency with which the formation of the MinE-ring and the MinD polar zones extended more than half of the cell length was higher in the C1 mutant (Table 2). Live cell imaging experiments spanning over an hour revealed that approximately 33.3% of cells (*n* = 45) showed peripheral MinD localization and no sign of disassembly. Another 35.6% of cells showed signs of disassembly from the extreme cell poles but appeared to stutter (Fig. 5B, 1; Movie S2). Interestingly, in approximately 31.1% of cells carrying the C1 mutant construct, we found no disassembly of the MinD polar zone once its growth reached midcell, i.e. the polar zone grew past the midcell until it reached the other end of the cell (Fig. 5B, 2 and 3; Movies S3 and S4), and the presence of the MinE ring did not stop its growth (Fig. 5B, 2). After the entire cell was peripherally displaying MinD fluorescence, disassembly re-initiated from the end of the cell where MinD fluorescence had resided the longest (Fig. 5B, 2 and 3). Western blot analysis showed no changes in the MinD to MinE ratio in cells (Fig. 5C), thus the aberrant movement of MinD and MinE was not a consequence of changing the intracellular concentration. We reasoned that when the MinD zone grows continuously past midcell, the cytosolic MinD available for maintaining basal exchange of MinD molecules between the cytosolic and membrane-bound pools gradually decreases. Therefore, dissociation gradually becomes dominant over association at the non-growing end, thus explaining why the initiation of disassembly of the MinD zone occurs at the extreme opposite pole of the cell. Furthermore, oscillation between the two poles was sometimes observed to follow an unusual movement of the MinD zone. These observations indicated that elevated MinD ATPase activity in the absence of the MinE–membrane interaction did not necessarily result in more efficient MinD disassembly. These results suggest that inefficient MinE–membrane interaction interferes with the ability of MinE to properly regulate oscillation cycles, thus proving the physiological significance of the direct MinE–membrane interaction.

**Table 2:** Colocalization patterns of Yfp-MinD and wild-type and mutant MinE-Cfp.

Localization pattern		MinE				
MinD polar zone	MinE ring	WT	R10G/ K11E/ K12E	K19E/ R21G	R29G/ R30G/ R31G	D45A/ V49A
≤ 0.5 cell length	+	55.0%	8.7%	0.0%	64.2%	0.3%
≤ 0.5 cell length	−	5.6%	9.9%	0.0%	1.8%	50.3%
≥ 0.5 cell length	+	8.3%	11.1%	0.0%	1.8%	0.0%
≥ 0.5 cell length	−	0.6%	35.1%	0.0%	0.0%	32.9%
Off cell pole	+/−	3.9%	0.7%	0.0%	4.2%	0.3%
Double polar zone	+/−	0.0%	3.5%	0.0%	0.0%	2.0%
Peripheral^a^	Dispersed^a^/focus^a^	20.6%	28.2%	100.0%	27.9%	6.9%
Not clear	Not clear	6.1%	2.8%	0.0%	0.0%	7.4%
*n*		180	425	506	159	392

a Please see Fig. 5A for peripheral (p), dispersed (d) and focus (f) patterns of localization.

Residues D45 and V49 in the C-terminal domain are known to be necessary for MinE ring formation and for arresting growth of the MinD polar zone at midcell (Shih *et al.*, 2002a). Here we demonstrated that the D45A/V49A mutant co-sedimented with liposomes less significantly than wild-type MinE (Fig. 4D) and was less capable of inducing membrane tubule formation (Fig. 4G). This inability to directly associate with the cell membrane may contribute to the loss of MinE ring formation in the D45A/V49A mutant. Additionally, this mutant appeared normal in its stimulation of the ATPase activity of MinD but interacted less well with MinD (Fig. 4A–C). All mutant phenotypes were similar to that of the C1 mutant, but unlike the C1 mutant directly affecting the membrane contact, the D45A/V49A mutant affects regulation conferred by the C-terminal domain. The results suggest that the C-terminal domain of MinE controls the interaction of the N-terminal domain of MinE with the membrane or with MinD.

The C2 mutation caused MinE to localize diffusely in the cytosol and this was accompanied by peripheral distribution of MinD in Δ*min* cells (Fig. 5A, Table 2). Additionally, we often found the C2 mutant MinE appeared in bright foci that may be aggregates of the overproduced and misfolded proteins. These foci could be found in minicells (Fig. 5A). This observation was consistent with our other results on the C2 mutant and further indicates that the C2 region is of crucial importance to all aspects of MinE function. We found that the C3 mutant did not affect formation of the MinD polar zone or the MinE ring (Fig. 5A, Table 2), but the oscillation rate seemed slower than with wild-type MinE. Here we used one half of an oscillation cycle as the unit to calculate the oscillation rate, because the observed rates varied between each half cycle. The rate of MinD oscillation in the presence of the C3 mutant was measured at 0.19 ± 0.1 half cycle per minute (*n* = 65 from 16 cells having more than two half cycles, cell length between 3.4 and 5.8 µm). This was significantly slower than the measured oscillation rate of the wild-type at 0.62 ± 0.21 half cycle per minute (*n* = 98 from 16 cells having more than two half cycles, cell length between 3.4 and 7.9 µm; Movie S1) in this study and the previously measured oscillation rate of 1.25 ± 0.24 full cycles per minute with wild-type MinE (Shih *et al.*, 2002a). The slower oscillation may also be due to the reduced MinD level in the cell based on the Western blot analysis.

In comparing the ability of the mutant MinE along with MinCD to complement a *min* deletion strain YLS1, we found all three MinE mutants failed to restore the wild-type division and caused cells to become long filaments (Fig. S4A). We also examined the abundance of the mutant proteins in these samples. While the abundance of the C2 and C3 mutants of MinE showed a comparable level to the wild-type, that of the C1 mutant was significantly reduced (Fig. S4B), indicating the C1 mutant protein was highly unstable in cells. We also expressed *minCD* and *minE* from separate promoters (P*_ara_* and P*_lac_*) in the same *min*-deletion background, but were unable to obtain a condition that could restore the wild-type division. In the *min* operon, the protein ratio of MinD and MinE is maintained through translational coupling (de Boer *et al.*, 1989). Since both promoters may show different levels of leaky expression in a population of cells and the control offered by P*_lac_* and P*_ara_* may be limited, it became difficult to restore the wild-type division in a population by reaching a balanced ratio between MinD and MinE using this approach. Therefore, the complementation results indicate the C2 and C3 mutants of MinE have lost their proper functions, but this approach is not adequate for assaying the function of the C1 mutant.

## Discussions

In this study we provide evidence that direct interaction between MinE and the membranes is a necessary factor for the localization and oscillation of MinDE. The N-terminal domain of MinE (MinE^1–31^) is responsible for the interaction that involves electrostatic attraction in the process. MinE^1–31^ carries eight positively charged residues among which the R10, K11 and K12 residues specifically function in membrane association and are not involved in stimulation of the MinD ATPase activity.

Electrostatic interaction is one of the mechanisms that mediate the peripheral membrane association of proteins (Mulgrew-Nesbitt *et al.*, 2006). The non-specific electrostatic interactions can contribute to protein targeting, orienting proteins, and lateral assembly of proteins on the membrane. Examples include the Src tyrosine kinase, the Endofin FYVE domain, and the retroviral Gag protein. However, it should be emphasized that electrostatic forces are often one of several mechanisms that take part in the observed membrane association of proteins. Additional forces such as hydrophobic interaction, and type and shape of the protein folds are often involved in stabilizing the protein–membrane interaction (Mulgrew-Nesbitt *et al.*, 2006). Our current study points at the N-terminal domain of MinE that can directly interact with the membrane through electrostatic forces, but the entire view and consequences of this interaction is yet to be fully elucidated.

In the earlier model explaining the molecular mechanism of the Min system, MinE recognized the midcell topological marker through its C-terminal domain (Zhao *et al.*, 1995). Based on our data presented here, we propose that the N-terminal domain of MinE confers topological specificity by binding to the membrane at the midcell zone that is enriched in negatively charged cardiolipin at certain stages during the cell cycle (Mileykovskaya and Dowhan, 2009). While cardiolipin shows fundamental importance in sustaining the MinE–membrane interaction, the phospholipid composition and the resulting net charge are potential factors that may regulate the interaction. The C-terminal domain of MinE sequesters the N-terminal domain in the cytosol, thus preventing MinE from contacting the cell membrane directly. This was reflected in only small amounts of the full-length MinE co-sedimenting with liposomes. After MinE is recruited by MinD to the membrane sites, the N-terminal domain of MinE becomes more accessible to the membrane, and an interaction ensues that is further stabilized by the high local concentration of anionic phospholipids. Self-organization of MinE through an unidentified mechanism delivered by the C-terminal domain may follow after the local concentration of membrane-bound MinE exceeds a threshold level, leading to formation of the MinE ring at midcell capping the medial edge of the MinD polar zone (Fig. 6A and B).

An interesting observation in this study is the ability of MinE to induce membrane deformation into membrane tubules *in vitro*. The function of this property may be explained by the C1 (R10G/K11E/K12E) mutant of MinE that was a specific membrane-binding mutant, accompanying elevated MinD ATPase activity and aberrant movement of MinDE in cells. A puzzling observation was that in the presence of the MinE ring, the MinD polar zone continued to grow past the ring (Fig. 5B, 2), indicating the MinE ring and ATP hydrolysis *per se* are not sufficient to switch on MinD polar zone disassembly. Therefore, the membrane-deformation function of MinE is likely a critical factor to initiate dissociation of MinD from the membrane (Fig. 6C). Membrane deformation caused by MinE may provide a physical force to strip MinD off the membrane at the onset of disassembly of the MinD polar zone.

A similar membrane-deforming property was identified for MinD through formation of the MinD helical filaments surrounding the tubules (Hu *et al.*, 2002), but the significance of the *in vitro* membrane deforming property of MinD in the Min system was unclear. Insertion of an amphipathic helix into one leaflet of the membrane can introduce changes in the local curvature of the membrane. Subsequent MinD polymerization upon associating with the membrane is likely to stabilize the changes in membrane curvature and further thrust membrane tubule formation. Unlike MinD, MinE shows no characteristic features of achieving membrane tubule formation. Our study indicated that the electrostatic interaction between the N-terminal domain of MinE and the membrane plays a role in the observed phenomenon. The N-terminal 22 residues of MinE are known to form an unstable helix structure in solution (King *et al.*, 1999) that may be a native state of the N-terminal domain. When the N-terminal domain is in contact with different interacting partners, such as MinD or the membrane, certain protein conformations may be stabilized to favour different interactions. The coat surrounding the membrane tubule may involve self-organization of MinE upon its binding to the membrane. The mechanism of this self-organization will require high resolution EM analysis. Meanwhile, the C-terminal domain of MinE serves as the candidate to control this self-organization process.

## Experimental procedures

### Bacterial strains and plasmids

The *E. coli* strain MC1000 [*araD*139 Δ(*araABC-leu*)*7679 galU galK*Δ(*lac*)X74 *rpsL thi*] (Casadaban and Cohen, 1980) was used for cloning. Strains BL21(DE3) [*F^-^ ompT hsdS*_B_(*r*_B_^-^*m*_B_^-^) *gal dcm* (DE3)]/pLysS and BL21(DE3) Δ*min* (Suefuji *et al.*, 2002) were used for protein overproduction. YLS1 (Shih *et al.*, 2005) was used in the fluorescence microscopy and the complementation assays. Cells were grown in Luria–Bertani (LB) medium supplemented with appropriate antibiotics. Plasmids pSOT13 [P*_T7_*::*minE-his*] and pSOT64 [P*_T7_*::*minE^D45A/V49A^-his*] were constructed by amplifying the *minE* and *minE^D45A/V49A^* gene fragments by PCR and cloning them into pHTPP13 (Shih *et al.*, 2002b) at the NcoI and XhoI sites. The construction of pSOT78 [P*_T7_*::*minE^32–88^-his*] was similar but the *minE^32–88^* fragment was cloned into pHTPP13 at the NdeI and XhoI sites. Plasmids pSOT93 [P*_T7_*::*minE^R10G/K11E/K12E^-his*], pSOT92 [P*_T7_*::*minE^K19E/R21G^-his*] and pSOT102 [P*_T7_*::*minE^R29G/R30G/R31G^-his*] were obtained by two-step site-directed mutagenesis to introduce substitutions at the desired sites. pSOT6 [P*_T7_*::*trx-his-minD*] was created by PCR amplification of the *minD* gene fragment and cloning it into pHTPP15 (Shih *et al.*, 2002b) at the EcoRI and XhoI sites. pMLB1113 (de Boer *et al.*, 1989) and pYLS97 [P*_lac_*::*yfp-minD*] (Shih *et al.*, 2003) were constructed as previously described. pSOT7 [P*_lac_*::*yfp*] was constructed by inserting the *yfp* fragment with a SD sequence into pMLB1113 at the EcoRI and HindIII sites. pSOT83 [P*_lac_*::*minE^1–31^-yfp*], pSOT109 [P*_lac_*::*minE^1–31(R10G/K11E/K12E)^-yfp*], pSOT110 [P*_lac_*::*minE^1–31(K19E/R21G)^-yfp*] and pSOT126 [P*_lac_*::*minE^1–31(R29G/R30G/R31G)^-yfp*] were constructed by PCR amplifying the *minE^1–31^* fragments from pYLS67 [P*_lac_*::*cfp-minD minE-yfp*] (Shih *et al.*, 2002a), pSOT93, pSOT92 and pSOT102, and re-ligating them into the large EcoRI-BamHI fragment of pYLS67. pSOT128 [P*_lac_*::*yfp-minD minE^R10G/K11E/K12E^-cfp*], pSOT129 [P*_lac_*::*yfp-minD minE^K19E/R21G^-cfp*] and pSOT130 [P*_lac_*::*yfp-minD minE^R29G/R30G/R31G^-cfp*] were generated by ligating the large XbaI-BamHI fragment of pYLS68 [P*_lac_*::*yfp-minD minE-cfp*] (Shih *et al.*, 2002a), the XbaI-XmnI *minD*-containing fragment of pYLS68 and the XmnI-BamHI fragments PCR amplified from pSOT93, pSOT92 and pSOT102 respectively.

pT25-*minD*[*cya^T25^-minD*] was constructed by ligating the *minD* fragment PCR amplified from pSY1083G (Fu *et al.*, 2001) into pT25 at the BamHI and KpnI sites. pT18-*minE*[*minE-cya^T18^*], pT18-*minE^1–31^*, pT18-*minE^32–88^*, pT18-*minE^D45A/V49A^*, pT18-*minE^R10G/K11E/K12E^*, pT18-*minE^K19E/R21G^* and pT18-*minE^R29G/R30G/R31G^* were generated by ligating the *minE* fragment that was PCR amplified from pSY1083G, pT18-*minE*, pSOT141, pSOT64, pSOT93, pSOT92 and pSOT102 respectively, into pT18 at the XhoI and HindIII sites.

### Overproduction and purification of MinE

Overnight cultures of BL21(DE3)/pLysS/pSOT13, pSOT78, pSOT64, pSOT93, pSOT92 or pSOT102 were diluted 20-fold into fresh LB medium supplemented with 0.4% glucose, 50 µg ml^−1^ kanamycin and 34 µg ml^−1^ chloramphenicol. The cultures were incubated at 30°C until the OD_600_ reached 0.6, at which point isopropyl–d-thiogalactoside (IPTG) was added to a final concentration of 0.5 mM and the culture further incubated at 30°C for a further 3 h. Cells were harvested and resuspended in a buffer containing 50 mM Tris-Cl pH 7.5 and 500 mM NaCl followed by three passages through a French press at 16 000 psi. The supernatant and insoluble fractions were separated by centrifugation at 61 000 *g* for 30 min at 4°C. The supernatant was subjected to affinity purification using His-select Nickel affinity gel (Sigma) and proteins were eluted with 250 mM imidazole. The crude purified MinE-His was then passed through a Hi-Load Superdex 75 column (GE Healthcare) and eluted in a buffer containing 50 mM Tris-Cl pH 7.5 and 5% glycerol. For long-term storage, the protein sample was added with 50% glycerol and preserved at −80°C. Samples were exchanged into 20 mM Tris-Cl pH 7.5, 200 mM sucrose (buffer A) for experiments. The wild-type MinE^1–31^ peptides were synthesized and prepared as a 1 mg ml^−1^ (270 µM) stock solution in buffer A. Protein samples were clarified by centrifugation at 21 000 *g* for 30 min or at 21 000 *g* for 5 min followed by filtering through 0.2 µm syringe filters to remove protein precipitates. The clarified samples were quantified before preparation of the reaction mixtures. Electrophoresis was done following standard protocols described in the Condensed Protocols from Molecular Cloning (Sambrook and Russell, 2006).

### Overproduction, purification and function of the MinD fusion protein

An overnight culture of BL21(DE3) Δ*min*/pSOT6 grown from a single colony was diluted 20-fold into fresh LB medium supplemented with 0.4% glucose and 50 µg ml^−1^ ampicillin. The culture was incubated at 30°C until the OD_600_ reached 0.6, when IPTG was added at a final concentration of 0.5 mM IPTG and incubated at 30°C for a further 3 h. Cells were harvested and resuspended in a buffer (50 mM Tris-Cl pH7.5, 500 mM NaCl) followed by three passages through a French press at 16 000 psi. The supernatant and insoluble fractions were separated by centrifugation at 61 000 *g* for 30 min at 4°C. The supernatant was subjected to affinity purification using His-select Nickel affinity gel (Sigma) and proteins were eluted with 20 mM imidazole. This crude purified Trx-His-MinD was treated with 0.72 unit thrombin/mg protein at 8°C for 1.5 h before being passed through a Sephacryl™ S200 column (GE Healthcare) and eluted in a buffer containing 20 mM Tris-Cl pH 7.5, 150 mM NaCl, 50 mM KCl and 5 mM MgCl_2_. For long-term storage at −80°C, the protein sample was exchanged into a buffer containing 50 mM HEPES pH 7.2, 150 mM KCl, 15 mM MgCl_2_, 0.1 mM EDTA, 5 mM DTT and 50% glycerol. Protein samples were clarified by centrifugation at 21 000 *g* for 30 min to remove protein precipitates and quantified before preparation of the reaction mixtures.

The resulting MinD fusion carried 40 additional amino acids (4.4 kDa) at its N-terminus. To ensure its function, we subcloned the gene fragment encoding the fusion protein (*trx'-minD minE*) into pMLB1113 (de Boer *et al.*, 1989) and the resulting plasmid (pSOT52) complemented the phenotype of a Δ*minDE* strain HL1 (Hale *et al.*, 2001) (Fig. S5). The complementation experiments were done following the procedures described previously (Shih *et al.*, 2005).

### Liposome preparation

The *E. coli* cell membrane contains three major types of phospholipids, including zwitterionic phosphatidylethanolamine (PE), anionic PG and CL in a ratio of approximately 7:2:1 (Mileykovskaya and Dowhan, 2005). *E. coli* lipids, including polar extract, PE, PG and CL were purchased from Avanti Polar Lipids, Ltd (USA) with PE : PG : CL ratio of 6.7:2.3:1 by weight, which equals a molar ratio of approximately 6.8:2.7:0.5. A 1 mg ml^−1^ phospholipid solution was prepared in chloroform and dried in a clean glass vial or a round-bottom flask followed by rehydration in buffer A. An extrusion method was used for preparation of large to small vesicles of uniform size, and a rotavapor was used to assist in preparation of giant vesicles. A detailed protocol is supplied in the supplemental data.

### Sedimentation assay

The purified proteins used in all experiments were clarified by centrifugation at 21 000 *g* for 30 min to remove precipitates and were quantified before use. MinE at 6 µM was incubated with 1 mg ml^−1^ liposomes (400 nm in diameter) in 90 µl buffer A for 60 min at 30°C. The samples were centrifuged at 21 000 *g* for 30 min at room temperature and divided into supernatant and pellet fractions. Supernatants and pellets were prepared in a sample buffer (12% glycerol, 5 mM Tris-Cl pH 6.8, 4% SDS, 2% β-mercaptoethanol and 0.01% serva blue), boiled for 5 min before separation on 12% Tris-Tricine-SDS gels (Schägger and von Jagow, 1987) that were then stained with Coomassie brilliant blue. Quantitative measurement of the band intensity was done using Multi Gauge Software (FujiFilm Corporation) and analysed in Microsoft Excel. The average and standard deviation of the band intensity was obtained from at least three independent experiments.

### Bacterial two-hybrid assay

Plasmids carrying *cya^T25^-minD* and *minE-cya^T18^* (wild-type or mutant) gene fusions were introduced into a *cya*^-^ strain of DHP1 [F^-^*glnV44(AS) recA1 endA1 gyrA96 (Nal^r^) thil hsdR17 spoT1 rfbD1*] (Karimova *et al.*, 1998). Fresh transformants were grown in LB medium supplemented with 0.5 mM IPTG, 100 µg ml^−1^ ampicillin and 34 µg ml^−1^ chloramphenicol overnight at 30°C. The overnight cultures were diluted 1:100 into fresh LB medium containing the same supplements and incubated at 30°C for an additional 8–9 h. Interaction between the two target proteins was reflected by the cAMP-inducible reporter gene *lacZ* encoding β-galactosidase that can be quantified as previously described (Pardee *et al.*, 1959). The results were converted into Miller units using the equation: 1000 × [OD_420_−(1.75 × OD_550_)/*t* × *v* × OD_600_], in which *t* is the reaction time in minutes and *v* is the volume of culture in millilitres used in each reaction. Four repeats of each combination were measured in each experiment and each combination was assayed at least three times. We expressed the final results as ‘relative interaction’ by normalizing to the wild-type MinD–MinE interaction (100%) in each independent set of experiments before statistical analysis.

For plate assays, fresh transformants were grown in LB medium supplemented with 0.4% glucose, 100 µg ml^−1^ ampicillin and 34 µg ml^−1^ chloramphenicol overnight at 30°C. The overnight cultures were diluted 10^4^- and 10^5^-fold in fresh LB before spotting 5 µl of each diluted culture on an LB agar plate supplemented with 0.5 mM IPTG, 40 µg ml^−1^ 5-bromo-4-chloro-3-indolyl-β-d-galactopyranoside (X-Gal), 100 µg ml^−1^ ampicillin and 34 µg ml^−1^ chloramphenicol. The plates were incubated at 30°C for 20–23 h before observation. Colony size and grade of the blue colour of the colony were the indicators of interaction.

### ATPase assay

The ATPase activity was measured using the malachite green phosphate assay kit (BioAssay Systems) according to the manufacturer's instructions. MinD (6 µM) and MinE (6 µM) were mixed in a reaction buffer (20 mM Tris-Cl pH 7.5, 50 mM KCl, 150 mM NaCl, 5 mM MgCl_2_) containing 0.5 mM ATP and 400 µg ml^−1^ liposomes that were 400 nm in diameter. The reactions were incubated at 37°C for 5 min before addition of the malachite green reagent. After colour development at room temperature for 10 min, the absorbance was recorded at 630 nm. The average and standard deviation of the activity was obtained from at least three independent experiments.

### Fluorescence microscopy

Cells for observing the localization of wild-type and mutant MinE^1–31^-Yfp were induced with 20 µM IPTG at 37°C for 60–90 min. Cells for the observation of cellular localization of Yfp-MinD and MinE-Cfp were grown as described previously (Shih *et al.*, 2005). An upright Olympus BX61 microscope was equipped with a PLAPON 60× (NA1.42) objective, Chroma ET-EYFP (49003), ET-CFP (49001), ET-mCherry (49008) and ET-GFP (49002) filter sets, a Hamamatsu Orca-AG cool Charge-Coupled Digital camera and Volocity (Improvision) for image acquisition and analysis.

To compare fluorescence profiles of membrane-associating MinE^1–31^-Yfp, Yfp-MinD and Yfp, we analysed 50 cells expressing each construct individually by measuring fluorescence intensity in an area of 5 pixels (approximately 0.54 µm) along the cell length and 49 pixels (approximately 5.32 µm) across the cell width. The fluorescence intensity was measured with NIH ImageJ as the average intensity of 5 pixels at each pixel position across the cell width. The data points between each measurement of 50 samples were aligned according to the cell position before obtaining the average fluorescence intensity. The first and last five values in each data set were used for background subtraction. For plotting the fluorescence distribution, we processed only 25 values covering the cell in each data set and normalized against the total fluorescence intensity (the sum of the 25 values). Calculation of kurtosis was used to describe the differences in cellular distribution of each construct. For calculation of the sample *G_2_*, we used standard formulas as described in Joanes and Gill (1998).

### Fractionation of cells expressing MinE^1–31^-Yfp and Western blot analysis

Cells were grown under the same conditions as for the microscopic examinations. Total cell lysate was obtained after resuspending the cell pellet from 100 ml culture in buffer A with 100 µM PMSF and disrupted by French Press. Low speed centrifugation (3200 *g*, 30 min) was performed to remove unbroken cells. The clear lysate was quantified and equal amounts (19.8 mg) of all clear lysates were brought to the same volume (9 ml) and ultracentrifuged for 100 min at 171 500 *g* at 4°C. The resulting pellet was dissolved in 100 µl 0.5% *n*-dodecyl-β-d-maltoside (DDM) in buffer A by incubating at 4°C with gentle shaking overnight. Before gel electrophoresis, the resuspended pellet fraction was clarified by centrifugation for 30 min at 27 000 *g* at 4°C. A 30 µl volume of the input fraction (66 µg), 30 µl of the corresponding supernatant after ultracentrifugation and 10 µl of the pellet fraction were separated on a 12% Tris-Tricine-SDS gel. Western blot analysis was performed according to the standard protocols described in Molecular Cloning (Sambrook and Russell, 2006). Monoclonal anti-Gfp antibody (Santa Cruz Biotechnology, sc-9996) was used for detecting the fusion proteins.

### Electron microscopy

MinE (6 µM) was mixed with a liposome suspension (0.4 mg ml^−1^ of 400 nm liposomes or 1 mg ml^−1^ giant liposomes) in buffer A. The reaction mixture (2.5–5 µl) was spotted onto a formvar/carbon- or carbon-coated copper grid (EMS or Pelco®) and incubated in a moist chamber for 10 min at room temperature. The sample was stained with 4% uranyl acetate for 30 s followed by washing and air-drying. EM images were collected using a Tecnai G2 Spirit TWIN electron microscope (FEI company) at 120 KeV equipped with a Gatan CCD Camera and processed by Digital Micrograph software (Version 3.9.2, Gatan). Alternatively, images were collected using a Hitachi H-700 electron microscope at 75 KeV equipped with an AMT XR40 CCD camera and processed by Digital Micrograph Software (Version 5.3, Advanced Microscopy Techniques). Adobe Photoshop and NIH ImageJ were used for further analysis and processing of the images.
