# Supplementary material for: Direct MinE–membrane interaction contributes to the proper localization of MinDE in E. coli
Source: Mol Microbiol. 2009 Dec 24;75(2):499–512. doi: 10.1111/j.1365-2958.2009.07006.x (PMC2814086; doi:10.1111/j.1365-2958.2009.07006.x)

Supplemental data for Direct MinE-membrane interaction contributes to the proper localization of MinDE in *E. coli*

Supplemental Experimental Procedures

Plasmids

pSOT138 (P_{lac}- *minC minD* $minE^{R10G/K11E/K12E}$), pSOT139 (P_{lac}- *minC minD* $minE^{K19E/R21G}$), and pSOT140 (P_{lac}- *minC minD* $minE^{R29G/R30G/R31G}$) were constructed in three fragment ligation reactions containing the *Eco*RI-*Hin*dIII fragment of pMLB1113, the *Eco*RI-*Xmn*I *minCD*-containing fragment from pSY1083 (P_{lac}- *minC minD* $minE^{WT}$; (Fu *et al.*, 2001)), and the *Xmn*I-*Hin*dIII *minE*-containing fragments from pSOT93, pSOT92, and pSOT102 respectively.

Liposome preparation

The glassware was washed with detergent and sonicated in ddH_2O, 95% EtOH, ddH_2O, acetone, ddH_2O, 1 M KOH, ddH_2O sequentially for 30 min each step. It was then stored in methanol and dried before use. For aliquoted phospholipids, we dissolved the dried phospholipids in chloroform and dispensed 1 or 2 mg into individual vials. After drying chloroform, argon was used to fill the vial before storing at -80°C for storage. To prepare giant liposomes, we dissolved 1 mg phospholipids in 1 mL chloroform before transferring to a round-bottom flask. The flask

was then connected to a rotavapor set at 100 rpm for drying in a 37°C waterbath until the chloroform completely evaporated. Buffer A (2 mL) was added to the flask to allow rehydration of the phospholipid bilayer. The flask was allowed to sit still in a beaker for 3 h. For preparation of liposomes of uniform size, we vacuum dried 2 mg phospholipids in a glass vial before adding 1 mL buffer A. The dried phospholipid was allowed to rehydrate for 1 h with gentle shaking every 15 min. The liposome solution was pushed through a filter with the desired pore size attached to an extruder 21 times. This protocol was modified from the information provided by the manufacturer (Avanti Polar Lipids, Inc., 'Liposome Preparation' [http://www.avantilipids.com/index.php?option=com_content&view=article&id=1383&Itemid=371]).

Mass spectrometry

The molecular weight of the MinE fusion protein was determined by a Q-TOF instrument (QSTAR XL, Applied Biosystems) in the positive mode. The data were deconvoluted by Analyst QS software (Applied Biosystems). To determine the molecular weight of proteins bound to the membranes (Mirza *et al.*, 2007), we extracted proteins from the pellet fraction in the sedimentation assay. The pellet collected from a 630 μL reaction was dissolved in 210 μL of chloroform and incubated at room temperature for 1 h with shaking before adding 210 μL

MeOH:H_2O (1:1 v/v) and vortexed for another 30 min. The mixture was centrifuged at 10,000 × g for 5 min and the lower layer was removed. The remaining mixture was further extracted by the addition of 1 mL chloroform and sonication for 30 min. It was centrifuged again and the lower layer was discarded. Proteins in the remaining aqueous phase and the interface materials were precipitated by the addition of four volumes of acetone, followed by incubation on ice for 1 h. After centrifugation at 10,000 × g for 5 min, the pellet was washed twice with acetone before dissolving in 30 μL 50 % acetonitrile containing 0.1 % formic acid for mass spectrometry analysis.

References

Crooks, G. E., G. Hon, J. M. Chandonia & S. E. Brenner, (2004) WebLogo: a sequence logo generator. *Genome Res* **14**: 1188-1190.

Fu, X., Y. L. Shih, Y. Zhang & L. I. Rothfield, (2001) The MinE ring required for proper placement of the division site is a mobile structure that changes its cellular location during the *Escherichia coli* division cycle. *Proc Natl Acad Sci USA* **98**: 980-985.

Mirza, S. P., B. D. Halligan, A. S. Greene & M. Olivier, (2007) Improved method for the analysis of membrane proteins by mass spectrometry. *Physiol Genomics* **30**: 89-94.

Notredame, C., D. G. Higgins & J. Heringa, (2000) T-Coffee: A novel method for fast and accurate multiple sequence alignment. *J Mol Biol* **302**: 205-217.

Supplemental Figures:

Fig. S1. Mass spectrometry analysis on the purified MinE fusion protein (A) and protein extracted from the pellet fraction of the sedimentation assay (B).

Analysis of the purified protein alone showed only one reliable molecular mass at 11,168.2 Da. Analysis of the sample containing the extracted protein showed two molecular masses at 11,168.1 and 22,336.1 Da hinting at the interaction of MinE with liposomes that may involve both the monomeric and dimeric forms of MinE.

Fig. S2. Sequence conservation and theoretical pI of MinE$^{1–31}$ from different bacterial species.

(A) The relative conservation at individual positions of MinE$^{1–31}$ was analyzed with T-Coffee (Notredame *et al.*, 2000) and displayed using WebLogo (Crooks *et al.*, 2004). MinE homologs were identified from 149 bacterial species and grouped into Gram-negative γ-proteobacteria, β-

proteobacteria, α-proteobacteria, Gram positive bacteria and cyanobacteria for presentation. The sites corresponding to the C1, C2, and C3 regions are labeled underneath each panel. **(B)** A table summarizing theoretical pI of MinE^{1-31} from different sources that fall in different ranges.

Fig. S3. MinE showed no endogenous ATPase activity.

The assays were performed as described in Fig. 4A except for the removal of MinD.

Fig. S4. Ability of various MinE mutants to complement the Δ*min* strain YLS1.

(A) The minicelling phenotype of YLS1 was restored by expression of $minCDE^{WT}$ from pSY1083 (P_{lac}- *minC minD* $minE^{WT}$) using induction by IPTG at concentrations ranging 10–25 μM. By replacing the wild-type MinE with the C1, C2, or C3 mutant of MinE, cells grew into a filamentous form when the concentration of IPTG used for induction was above 10 μM. Scale bar: 10 μm. **(B)** Western blot analysis on cells harvested from the complementation assays. A range of IPTG concentrations (0–100 μM) was tested for induction. In this experiment, equal amounts of the total cell lysates (25 μg) were separated on Tris-Tricine-SDS gels followed by Western blotting. While the C2 and C3 mutants demonstrated protein levels comparable to the wild-type MinE, the C1 mutant appeared less abundant and the expression level could not be restored close to the wild-type level by induction with higher concentrations of IPTG. Thus this

complementation assay was not an adequate method for assaying the function of the C1 mutant. Note that all three mutant MinE proteins ran at lower positions compared with the wild-type MinE, this may be due to the charge difference in these MinE proteins.

Fig. S5. Ability of the MinD fusion protein to complement the Δ*minDE* strain HL1. DIC images of **(A)** HL1/pMLB1113 [*ΔminDE/*empty plasmid] and **(B)** HL1/pSOT52 [*ΔminDE/*P_{lac}::*trx'-minD minE*] that were grown in the presence of 15 μM IPTG.

Supplemental Movies

Movie 1. Pole-to-pole oscillation of MinD in strain YLS1/pYLS68 (Δ*minCDE*/P_{lac}::*yfp-minD minE-cfp*). The image sequence was taken at 15 s intervals. The movie was played at a speed of 10 frames per second (fps).

Movie 2. Stuttered movement of MinD peripheral fluorescence from one end of a cell (strain YLS1/pSOT128 [Δ*minCDE*/P_{lac}:: *yfp-minD* $minE^{R10G/K11E/K12E}$*-cfp*]). This movie shows the same cell as presented in Fig. 5B 1. The image sequence was taken at 15 s intervals. The movie was made from every third image and was played at 10 fps.

Movie 3. Aberrant movement of the MinD polar zone in strain YLS1/pSOT128 [Δ*minCDE*/P_{lac}:: *yfp-minD minE*$^{R10G/K11E/K12E}$*-cfp*]). This movie shows the same cell as presented in Fig. 5B 2. The image sequence was taken at 15 s intervals. The movie was played at 10 fps.

Movie 4. Aberrant movement of the MinD polar zone in strain YLS1/pSOT128 [Δ*minCDE*/P_{lac}:: *yfp-minD minE*$^{R10G/K11E/K12E}$*-cfp*]). This movie shows the same cell as presented in Fig. 5B 3. The image sequence was taken at 15 s intervals. The movie was compiled from every fourth image and played at 10 fps.

Fig. S1

(A)

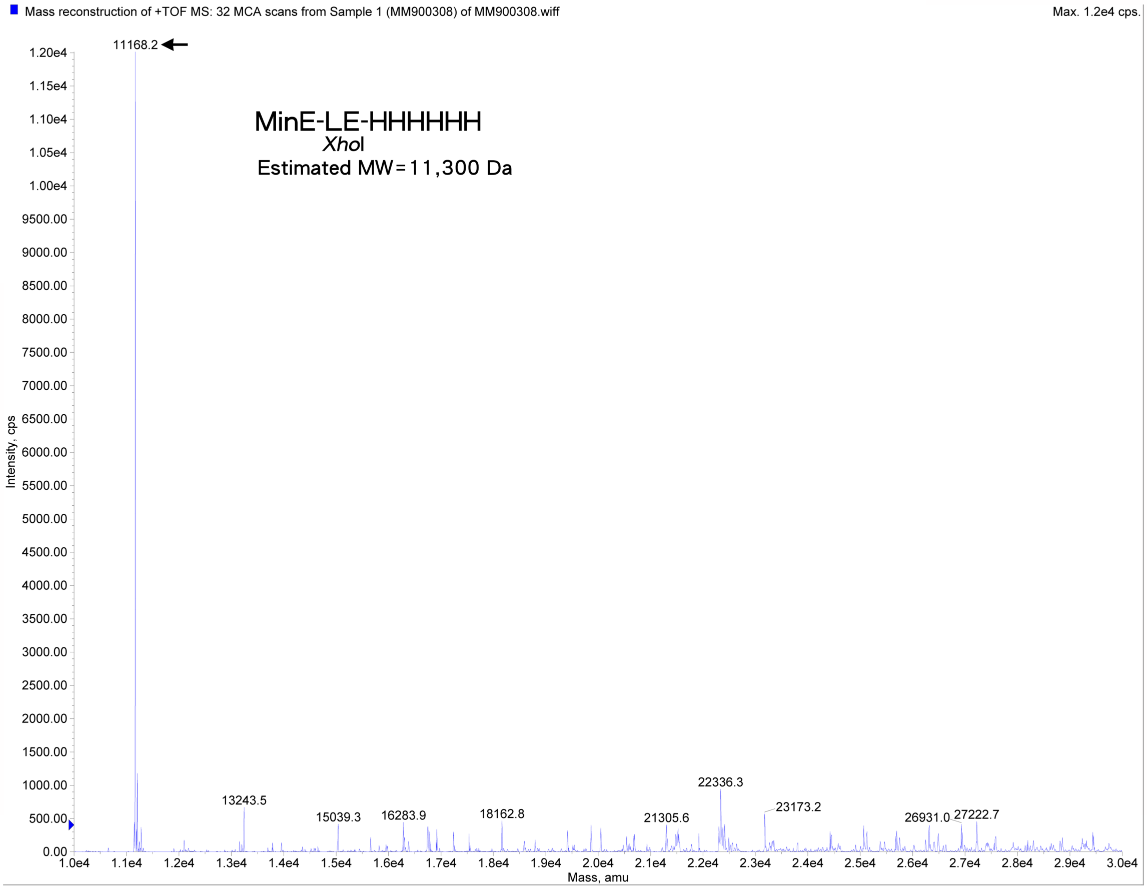

Mass reconstruction of +TOF MS: 32 MCA scans from Sample 1 (MM900308) of MM900308.wiff
Max. 1.2e4 cps.
11168.2
MinE-LE-HHHHHH
XhoI
Estimated MW=11,300 Da
13243.5
15039.3
16283.9
18162.8
21305.6
22336.3
23173.2
26931.0
27222.7
Intensity, cps
Mass, amu

(B)

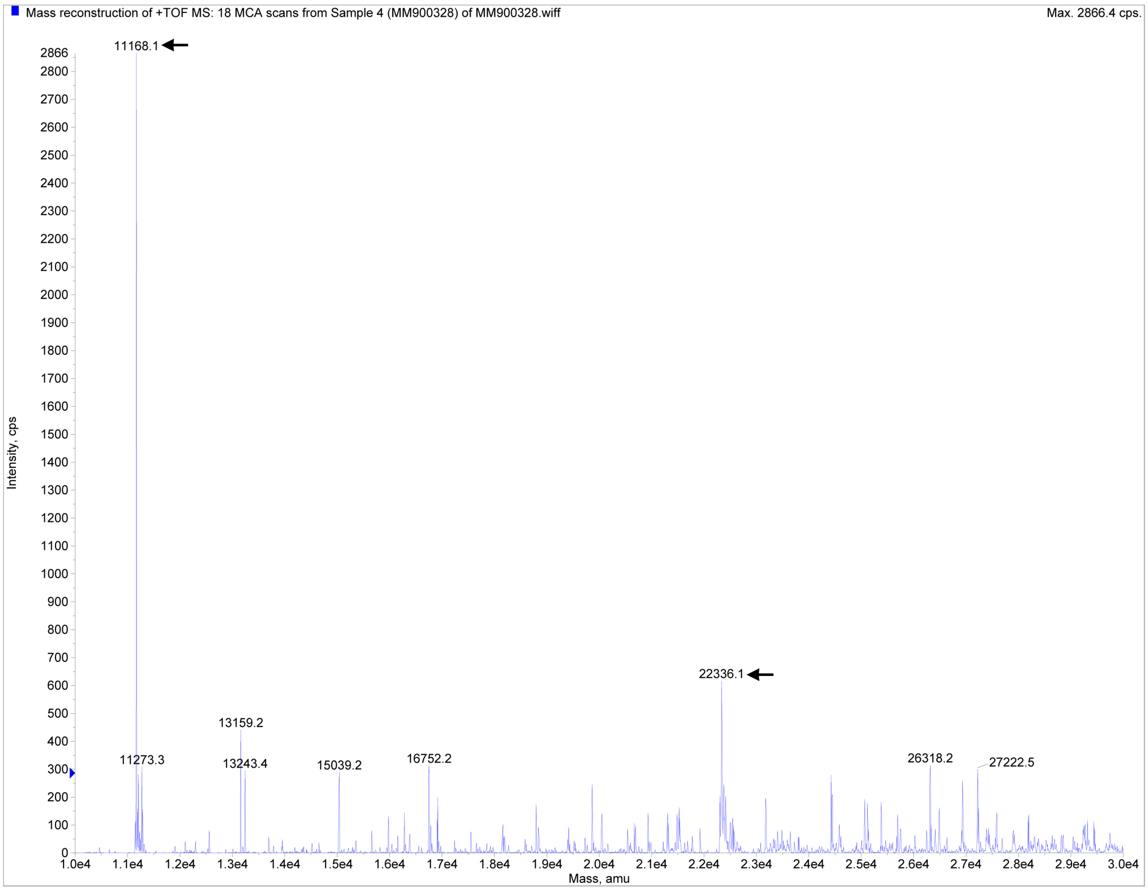

Mass reconstruction of +TOF MS: 18 MCA scans from Sample 4 (MM900328) of MM900328.wiff
Max. 2866.4 cps.
11168.1
11273.3
13159.2
13243.4
15039.2
16752.2
22336.1
26318.2
27222.5
Intensity, cps
Mass, amu

Fig. S2

(A)

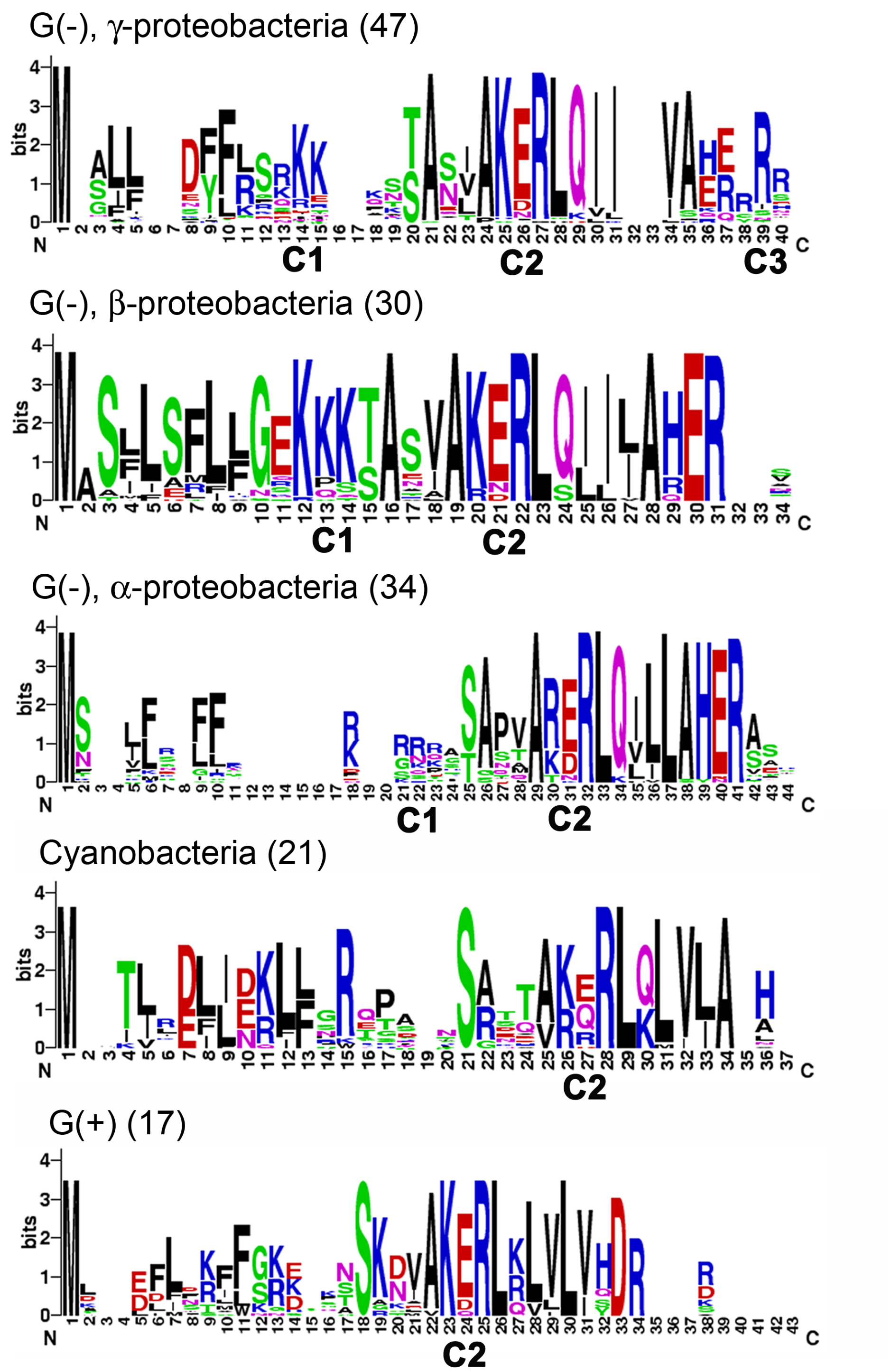

(B)

Source \ Theoretical pI of MinE$^{1-31}$		≤ 8.99	9 - 9.99	10 - 10.99	11 - 11.99	≥ 12	*total*
Gram (-)	γ-proteobacteria	5	11	10	20	1	47
	β-proteobacteria	0	17	11	2	0	30
	α-proteobacteria	0	4	4	24	2	34
Cyanobacteria		1	5	12	3	0	21
Gram (+)		1	9	6	1	0	17
total		7	46	43	50	3	149

Fig. S3

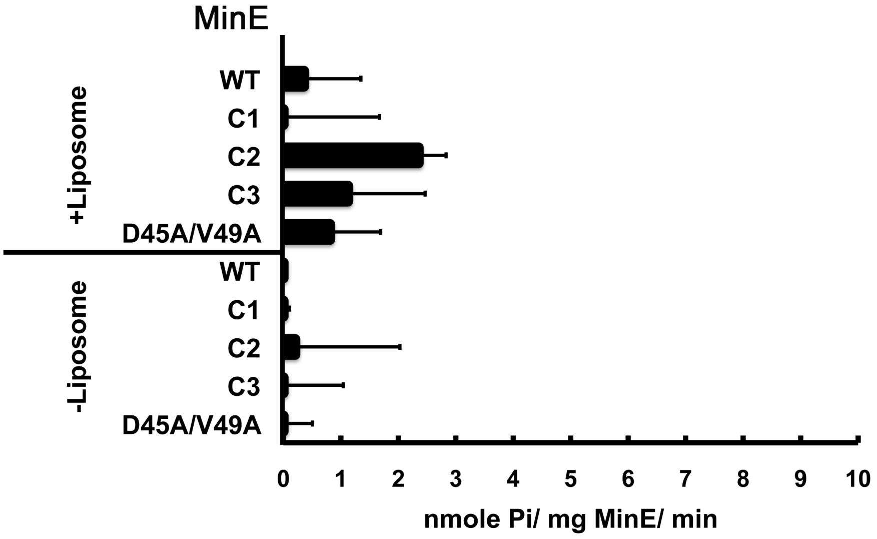
MinE
+Liposome
WT
C1
C2
C3
D45A/V49A
-Liposome
WT
C1
C2
C3
D45A/V49A
0
1
2
3
4
5
6
7
8
9
10
nmole Pi/ mg MinE/ min

Fig. S4

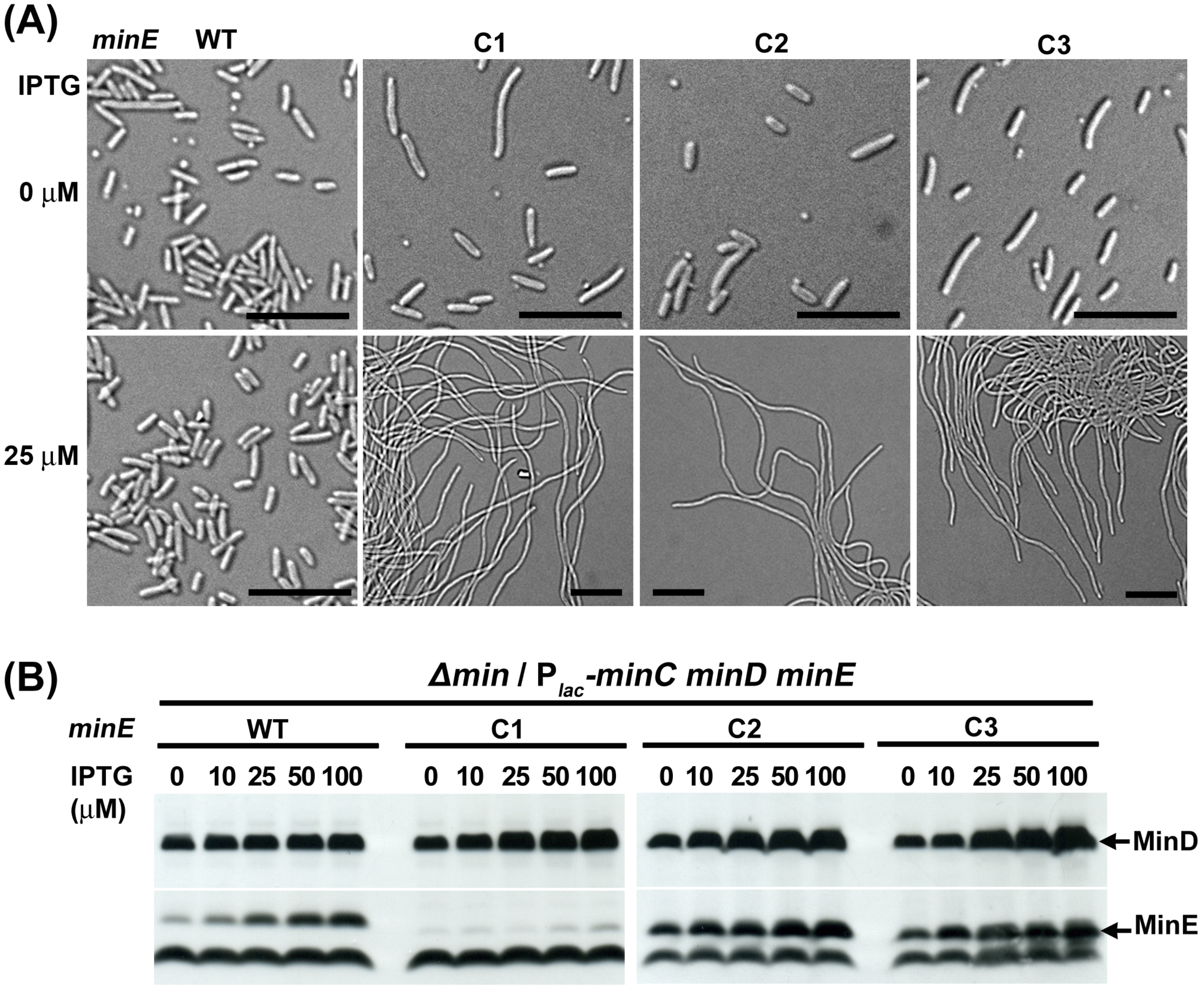
(A)
minE
WT
C1
C2
C3
IPTG
0 μM
25 μM
(B)
Δmin / P lac -minC minD minE
minE
WT
C1
C2
C3
IPTG (μM)
0 10 25 50 100
0 10 25 50 100
0 10 25 50 100
0 10 25 50 100
MinD
MinE

Fig. S5

(A) *ΔminDE*/ empty plasmid

(B) *ΔminDE*/ P_{lac}::*trx'-minD minE*

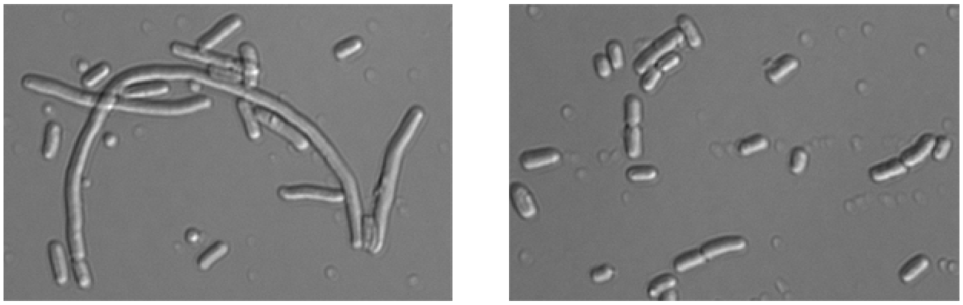

Supplement: Supplementary file 1 [file mmi0075-0499-SD1.pdf]
